# Therapeutical progress in sepsis-induced cardiomyopathy

**DOI:** 10.3389/fcvm.2026.1805393

**Published:** 2026-04-02

**Authors:** Jinbo Zhang, Shiying Sheng, Weiwei Luo, Zhaocai Zhang

**Affiliations:** 1 Emergency Intensive Care Unit, The First People’s Hospital of Wenling, Wenling, Zhejiang, China; 2 Department of Critical Care Medicine, Second Affiliated Hospital, Zhejiang University School of Medicine, Hangzhou, Zhejiang, China

**Keywords:** pathogenesis, repurposed drugs, sepsis-induced cardiomyopathy, traditional Chinese medicine, treatment

## Abstract

Sepsis-induced cardiomyopathy (SCM) is a life-threatening complication of severe sepsis with a high mortality rate. This review comprehensively explores SCM. It details the multifaceted pathogenesis, including inflammatory storm, mitochondrial dysfunction, abnormal calcium handling, complement activation, and emerging mechanisms related to exosomes and non-coding RNAs. We propose an integrated mechanistic model centered on the “energy metabolism-calcium handling” axis to explain the unique reversibility of SCM. Conventional treatments like antibiotic therapy, fluid management, and the use of vasopressors and inotropic agents are discussed, along with their limitations. Promising strategies such as repurposing old drugs, applying traditional Chinese medicine, and new approaches are presented. To bridge the gap between mechanistic understanding and clinical application, we categorize these emerging therapies according to the primary pathological pathway they target: inflammation, mitochondrial dysfunction, or calcium dysregulation. Furthermore, we introduce a framework for phenotype-guided treatment and critically evaluate the level of clinical evidence for each intervention. Small active molecules and nanomedicine also show potential in SCM treatment. Future research should focus on large-scale clinical trials to validate these therapies and integrate precision medicine strategies for better patient outcomes.

## 1 Introduction

Sepsis-induced cardiomyopathy (SCM), a life-threatening complication of severe sepsis characterized by reversible myocardial dysfunction, remains a major challenge in critical care medicine. Despite advances in supportive therapies, the mortality rate of SCM patients exceeds 50% due to the intricate pathogenesis involving exaggerated inflammation, mitochondrial dysfunction, calcium handling disorders, and complement activation (1). Conventional treatments, including antibiotics, fluid management, and vasoactive agents, primarily focus on symptom alleviation rather than addressing the underlying molecular mechanisms, leading to limited efficacy in improving cardiac recovery and long-term outcomes (2, 3). To bridge this therapeutic gap, repurposing existing drugs with established safety profiles and exploring traditional Chinese medicine (TCM) have emerged as promising strategies (4–6). This review systematically summarizes the pathophysiological mechanisms of SCM, evaluates conventional treatments, and discusses recent advancements in drug repurposing and TCM-based interventions. Crucially, we aim to synthesize these elements into a cohesive framework that connects specific pathological pathways to targeted therapies, considers patient heterogeneity, and assesses the strength of current evidence, thereby providing novel and actionable insights for precision therapy in SCM management.

## 2 Pathogenesis of septic cardiomyopathy

The pathogenesis of SCM is multifaceted and intricate, serving as the foundation for developing treatment strategies. This condition involves various mechanisms that contribute to its complexity.

### 2.1 Towards an integrated model of reversible myocardial depression in SCM

The clinical hallmark of SCM is its reversibility, which suggests a state of profound but non-structural cellular dysfunction, often termed “myocardial stunning.” The mechanisms described below do not act in isolation but are tightly interconnected, creating a vicious cycle that culminates in reversible cardiac failure. The central, unifying theme is an acute energy crisis coupled with disrupted calcium homeostasis.

The initiating event is the “inflammatory storm.” Pathogen- and damage-associated molecular patterns (PAMPs/DAMPs) trigger an overwhelming release of cytokines like TNF-α and IL-1β. These cytokines have a dual effect: they directly impair cardiomyocyte function and, more importantly, induce severe “mitochondrial dysfunction” by increasing reactive oxygen species (ROS) production and opening the mitochondrial permeability transition pore (mPTP). This leads to a catastrophic failure in oxidative phosphorylation and a sharp decline in ATP production.

This energy deficit directly impacts the machinery of “calcium handling.” The sarcoplasmic reticulum calcium-ATPase (SERCA2a), which is responsible for sequestering calcium to induce relaxation, is highly ATP-dependent. With insufficient ATP, SERCA2a function fails. Concurrently, cytokine signaling can inhibit the L-type calcium channels, reducing calcium influx. The combination of reduced calcium influx and impaired reuptake results in abnormal intracellular calcium transients. This manifests clinically as both systolic dysfunction (due to poor myofilament activation) and diastolic dysfunction (due to impaired relaxation).

Thus, SCM can be conceptualized as a condition where an initial inflammatory insult triggers mitochondrial failure, which in turn starves the calcium cycling machinery of energy, leading to a state of reversible contractile failure. This model explains why therapies targeting any point in this axis-inflammation, mitochondrial function, or calcium handling-could potentially restore cardiac function, provided the structural integrity of the cardiomyocyte is preserved, which underpins the condition's reversibility.

### 2.2 Inflammatory storm

An exaggerated inflammatory response triggered by sepsis plays a crucial role in the progression of SCM. Pathogen-associated molecular patterns (PAMPs), such as lipopolysaccharide (LPS) in Gram-negative bacteria, activate immune cells by binding to pattern-recognition receptors (7). This activation triggers downstream signaling pathways, leading to the production and release of pro-inflammatory cytokines like tumor necrosis factor-α (TNF-α), interleukin-1β (IL-1β), and interleukin-6 (IL-6). These cytokines directly harm cardiomyocytes, induce apoptosis, and inhibit myocardial contractility. TNF-α can down-regulate the expression of cardiac myofilament proteins and sarcoplasmic reticulum calcium-ATPase, disrupting calcium handling and weakening myocardial contractility. Additionally, damage-associated molecular patterns (DAMPs), such as high-mobility group box 1 protein (HMGB1), are released during sepsis and promote the infiltration of inflammatory cells into the myocardium, exacerbating cardiac inflammation (8).

### 2.3 Mitochondrial dysfunction

Mitochondria, the energy producers in cardiomyocytes, are impaired during sepsis. Factors like reactive oxygen species (ROS) overproduction can damage mitochondrial membranes, leading to the opening of mitochondrial permeability transition pores (mPTPs). This disrupts the mitochondrial membrane potential, affects oxidative phosphorylation, and reduces ATP production, leaving cardiomyocytes energy-starved and compromising myocardial contractility. Mitochondrial dysfunction also triggers the release of cytochrome c, activating the apoptotic pathway and accelerating cardiomyocyte apoptosis (9).

### 2.4 Abnormal calcium handling

Calcium handling disorders occur in SCM, disrupting the precise regulation of intracellular calcium ion concentration, which are necessary for maintaining normal myocardial contractile and relaxant function (10). Pro-inflammatory cytokines and other factors can affect the function of calcium channels and transporters in cardiomyocytes. For instance, L-type calcium channel activity may be inhibited, resulting reduced calcium influx during membrane depolarization. Additionally, the function of the sarcoplasmic reticulum calcium-ATPase, which is responsible for re-sequestering calcium during relaxation, may also be impaired. These disturbances lead to abnormal intracellular calcium dynamics, diminished myocardial contractility, and impaired relaxation (11).

### 2.5 Complement activation

The complement system, a vital component of the innate immune system, is activated in sepsis through classical, alternative, and lectin pathways. Activated complement components form membrane attack complexes (MACs) directly damage cardiomyocyte membranes, causing cell lysis and death. Moreover, complement activation products like C3a and C5a are potent inflammatory mediators, promoting the recruitment and activation of neutrophils and macrophages, thereby intensifying the local inflammatory response in the myocardium and exacerbating myocardial injury (12).

### 2.6 Emerging mechanisms

Recent studies have identified the role of exosomes and non-coding RNAs (ncRNAs) in the pathogenesis of SCM. Exosomes, small extracellular vesicles carrying bioactive molecules like proteins, nucleic acids, and lipids, are secreted by immune cells and cardiomyocytes during sepsis (13). Exosomes from immune cells may carry pro-inflammatory cytokines and microRNAs (miRNAs) that can be transferred to cardiomyocytes, exacerbating inflammation and myocardial injury. Conversely, exosomes from mesenchymal stem cells (MSCs) can deliver anti-inflammatory and cardioprotective factors like miR-125b and miR-146a, attenuating inflammation and oxidative stress and promoting myocardial repair. Non-coding RNAs (ncRNAs), including miRNAs and long non-coding RNAs (lncRNAs), are critically involved in the regulation of gene expression and are frequently dysregulated in the pathophysiological context of SCM (14, 15). For example, up-regulated miR-155 targets inflammation and apoptosis-related genes, while down-regulated miR-1 has a cardioprotective effect. LncRNAs like MALAT1 and H19 are also involved in the pathogenesis of SCM, regulating inflammation, apoptosis, and interacting with miRNAs (16). Studying the role of ncRNAs in SCM provides valuable insights into the molecular mechanisms of the disease and highlights their potential as novel diagnostic biomarkers and therapeutic targets.

## 3 Therapeutic strategies for septic cardiomyopathy

To provide a clear roadmap for clinicians and researchers, we have categorized the therapeutic landscape of SCM based on the primary pathological mechanism they address. This approach links each intervention directly to the biological problem it aims to solve, as outlined in our integrated model (Section 2.1). Furthermore, we present a summary of the level of clinical evidence for these strategies in Table 1.

**Table 1:** Evidence levels for therapeutic strategies in sepsis-induced cardiomyopathy.

Therapeutic category	Specific agent/Therapy	Primary target/Mechanism	Evidence level	Key references
Conventional care	Antibiotics & source control	Pathogen eradication	Standard of care	(17)
	Fluid management	Hemodynamic support	Standard of care	(18)
	Norepinephrine	Vasopressor (MAP support)	Standard of care	(19)
	Dobutamine/Milrinone	Inotrope (CO support)	Standard of care	(20, 21)
	Levosimendan	Calcium sensitizer	Clinical (RCTs)	(22)
	Mechanical support (ECMO/IABP)	Circulatory support	Clinical (case series/registry)	(23, 24)
Repurposed drugs	Statins	Anti-inflammatory (NF-κB inhibition)	Clinical (observational)/Pre-clinical	(25, 26)
	β-Blockers (e.g., Esmolol)	Anti-adrenergic/Anti-inflammatory	Clinical (small RCTs)/Pre-clinical	(27, 28)
	Vitamin C	Antioxidant/Anti-inflammatory	Clinical (RCTs with conflicting results)	(29–31)
	Melatonin	Antioxidant/Anti-inflammatory	Pre-clinical	(32, 33)
	Metformin	AMPK activation/Metabolic modulation	Pre-clinical	(34–37)
	ACEIs/ARBs	RAAS modulation	Pre-clinical	(38–40)
Traditional chinese medicine	Emodin	Anti-inflammatory (NF-κB)/Antioxidant	Pre-clinical	(41–44)
	Artemisinin	Anti-inflammatory/Immunomodulatory	Pre-clinical	(45, 46)
	Shenfu injection (SFI)	Anti-apoptotic (AKT/GSK-3β)	Pre-clinical	(47, 48)
Novel approaches	Corticosteroids	Broad anti-inflammatory	Clinical (Guidelines/Controversial)	(49)
	Anti-cytokine antibodies (e.g., Anti-TNF-α)	Specific anti-inflammatory	Clinical (RCTs - negative/unconvincing)	(50)
	Mitochondrial-targeted antioxidants (e.g., MitoQ)	Mitochondrial protection	Pre-clinical	(51)
	Mesenchymal stem cell (MSC) therapy	Immunomodulation/Paracrine effects	Pre-clinical	(52, 53)
Active small molecules	Resveratrol	Sirt1 activation/Anti-ferroptosis	Pre-clinical	(54–56)
	N-acetylcysteine (NAC)	Antioxidant (Glutathione precursor)	Pre-clinical	(57)
Nanomedicine	Liposomes, polymeric NPs	Targeted drug delivery	Pre-clinical	(58–63)
	Cerium oxide nanoparticles (Nanoceria)	Antioxidant/Anti-inflammatory	Pre-clinical	(64–67)

### 3.1 Conventional treatments for SCM

The conventional treatment of SCM involves a multifaceted approach (2), including timely antibiotic therapy, careful fluid management, and the strategic use of vasopressors, inotropic agents, and mechanical circulatory support devices, all aimed at improving clinical outcomes and facilitating myocardial recovery (17).

#### 3.1.1 Antibiotic therapy and source control

**Figure 1:**
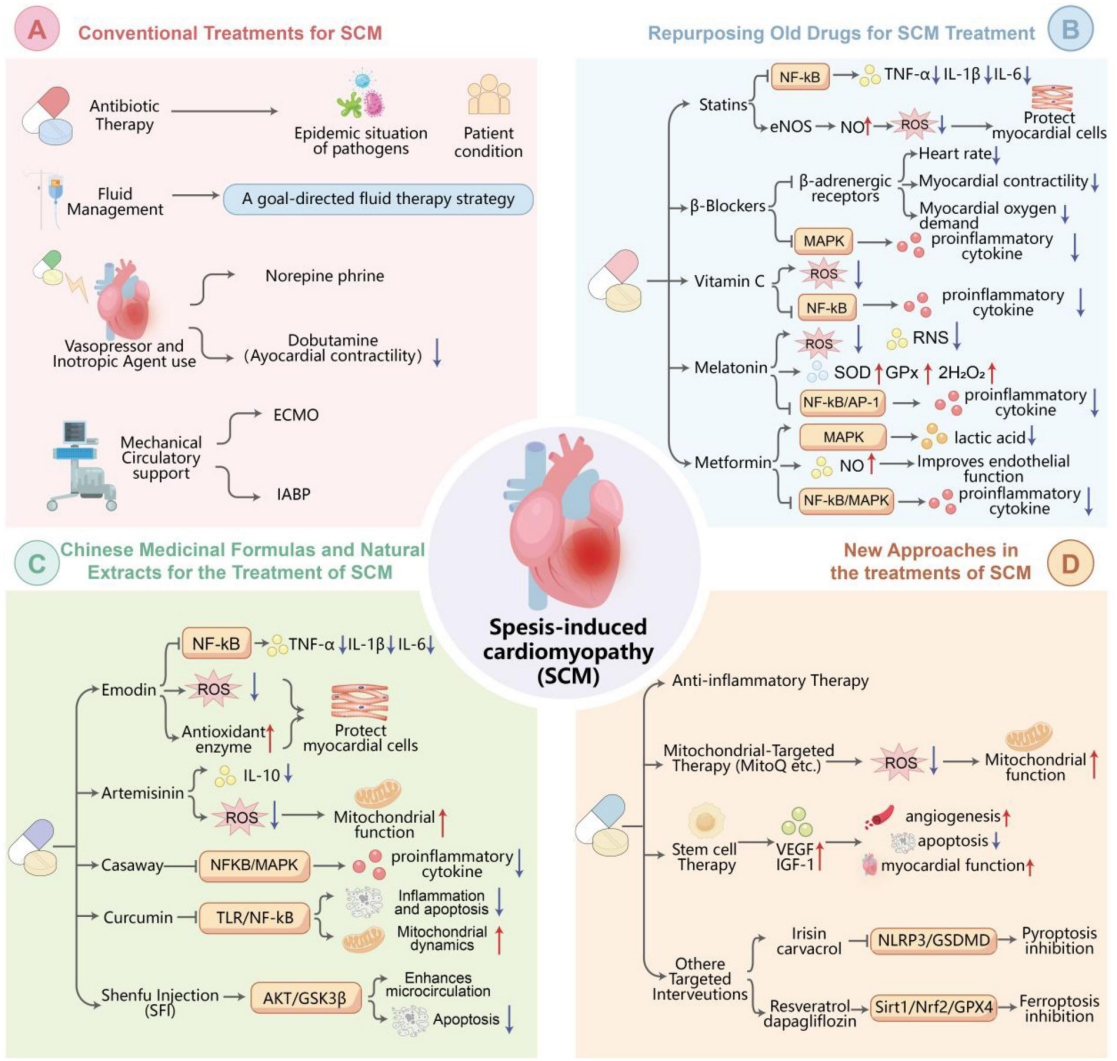
Therapeutic strategies for septic cardiomyopathy. (**A**) Conventional treatments include antibiotic therapy, fluid management, vasopressors (e.g., norepinephrine), and inotropic agents (e.g., dobutamine). (**B**) Repurposed drugs such as statins, β-blockers, vitamin C, metformin and metabolism. (**C**) Traditional Chinese Medicine components like emodin, artemisinin,casaway,curcumin and Shenfu Injection (SFI). (**D**) Novel approaches encompass mitochondrial-targeted therapy, Anti-inflammatory Therapy, stem cell therapy, and inhibition of pyroptosis or ferroptosis.

Prompt administration of broad-spectrum antibiotics is crucial once sepsis is suspected, based on local pathogen prevalence and the individual patient's clinical status. Subsequently, antibiotic therapy should be de-escalated to narrow-spectrum agent based on pathogen culture and drug sensitivity test results. In addition to antibiotics, effective source control measures, including abscess drainage, removal of infected foreign bodies, and resection of necrotic tissue are essential to reduce pathogen burden and toxin release. These interventions mitigate the inflammatory response and protect against myocardial injury (17) (see Figure 1A).

#### 3.1.2 Fluid management

Appropriate fluid management is vital to maintain effective circulating blood volume, improve tissue perfusion, and ensure adequate myocardial oxygen supply. A goal-directed fluid therapy strategy is recommended, incorporating dynamic monitoring of hemodynamic parameters such as Central Venous Pressure (CVP), Pulmonary Artery Wedge Pressure (PAWP) and Cardiac Output (CO) to guide the rate and volume of fluid infusion. Initial rapid fluid resuscitation is needed to correct hypovolemia, followed by careful titration to prevent fluid overload (18) (see Figure 1A).

#### 3.1.3 Vasopressor and inotropic agent use

When fluid resuscitation is inadequate to maintain Mean Arterial Pressure (MAP), vasopressors like norepinephrine (the first-line agent) are used to increase peripheral vascular resistance and blood pressure. Vasopressin can be used as an adjunct to norepinephrine for additional vasoconstrictive effects (19).

In patients with significant impairment of myocardial contractility, inotropic agents such as dobutamine (β1-adrenergic receptor agonist), milrinone (phosphodiesterase inhibitor) and levosimendan (calcium-sensitizing agent) can be considered to improve myocardial contractility and cardiac output (20, 21). However, continuous electrocardiogram (ECG) monitoring and close hemodynamic surveillance are required to optimize therapeutic efficacy while minimizing potential adverse effects. Levosimendan binds to troponin C in cardiomyocytes, increasing the sensitivity of troponin C to calcium ions without elevating the intracellular calcium concentration. This mechanism enhances myocardial contractility while minimizing the energy expenditure associated with calcium-dependent contraction. In SCM patients with diminished myocardial contractility, levosimendan can improve cardiac output and hemodynamic stability, offering a valuable therapeutic option for mitigating heart failure-like symptoms (22) (see Figure 1A).

#### 3.1.4 Mechanical circulatory support

Extracorporeal Membrane Oxygenation (ECMO): ECMO provides temporary circulatory and respiratory support, allowing the heart to rest and recover. Venous-arterial (VA) ECMO can support cardiac function by providing additional blood flow and oxygenation. However, strict patient selection and careful management are required due to potential complications like bleeding, thrombosis, and infection (23, 24) (see Figure 1A).

Intra-aortic Balloon Counterpulsation (IABP): IABP is placed in the descending aorta to improve coronary blood flow and reduce left ventricular afterload. Due to its relatively ease of operate and lower complication rate compared to ECMO, IABP is suitable for patients with mild to moderate cardiac impairment. However, its effectiveness in cases of severe SCM is limited (23) (see Figure 1A).

### 3.2 Pathway-targeted and repurposed drugs for SCM treatment

The repurposing of existing drugs, which involves identifying new therapeutic applications for medicines already approved for other conditions, has become an appealing strategy in the treatment of SCM. Given that these drugs often have well-characterized safety profiles, known pharmacokinetics, and lower development costs, they represent promising candidates for SCM therapy. Here, we organize these drugs by their primary mechanism of action in the context of the integrated SCM model.

#### 3.2.1 Anti-inflammatory agents

##### 3.2.1.1 Statins

Originally developed as lipid-lowering agents, statins have demonstrated multiple benefits in SCM treatment. Their therapeutic potential in SCM is primarily attributed to their anti-inflammatory and antioxidant properties. Statins inhibit the activation of nuclear factor-κB (NF-κB), a key transcription factor in the inflammatory cascade. By reducing the prenylation of small G-proteins, statins prevent the translocation of NF-κB into the nucleus, thereby decreasing the expression of pro-inflammatory cytokines like tumor necrosis factor-α (TNF-α), interleukin-1β (IL-1β), and interleukin-6 (IL-6). Additionally, statins enhance the expression of endothelial nitric oxide synthase (eNOS), leading to increased production of nitric oxide (NO). NO has vasodilatory effects and acts as an antioxidant, scavenging reactive oxygen species (ROS) and protecting cardiomyocytes from oxidative damage (25). Clinical studies have suggested that statin therapy in sepsis patients may be associated with a lower incidence of SCM and improved survival rates (26). However, further large-scale, randomized controlled trials are necessary for conclusive evidence (see Figure 1B).

##### 3.2.1.2 β-Blockers

β-Blockers, commonly used to treat cardiovascular diseases like hypertension and heart failure, can also play a role in SCM treatment. In sepsis, the sympathetic nervous system is overactivated, increasing myocardial oxygen consumption and potentially causing arrhythmias. β-Blockers block β-adrenergic receptors, reducing excessive catecholamine stimulation of the heart. This helps to decrease heart rate, myocardial contractility, and myocardial oxygen demand, which is beneficial for protecting the heart under stress. Moreover, β-Blockers have been shown to inhibit the mitogen-activated protein kinase (MAPK) signaling pathway and suppress pro-inflammatory cytokine production, thereby attenuating the systemic inflammatory response in SCM (27). Small-scale clinical studies have suggested that carefully titrated use of β-blocker in SCM patients may improve cardiac function and clinical outcomes, but optimal dosing and patient selection criteria require further exploration (28) (see Figure 1B).

#### 3.2.2 Antioxidants and mitochondrial protectants

##### 3.2.2.1 Vitamin C

Vitamin C, a well-known antioxidant, has potential in SCM treatment due to its antioxidant effect in reducing oxidative stress, which is prominent in SCM (29). Vitamin C directly scavenges ROS like hydroxyl radicals and hydrogen peroxide, protecting cardiomyocyte membranes, proteins, and DNA from oxidative damage (30). It also has anti-inflammatory properties, inhibiting NF-κB activation and reducing pro-inflammatory cytokine production, like statins (25). Clinical trials, such as the VICTAS trial, have investigated the combination of vitamin C, thiamine, and hydrocortisone in sepsis patients, showing potential benefits in improving organ function and reducing mortality, though the specific contribution of vitamin C to SCM improvement needs more research (30, 31) (see Figure 1B).

##### 3.2.2.2 Melatonin

Melatonin, primarily known for regulating sleep-wake cycles, exhibits multiple benefits in SCM. It is a powerful antioxidant and anti-inflammatory agent. Melatonin directly scavenges ROS and reactive nitrogen species (RNS) and up-regulates the expression of antioxidant enzymes like superoxide dismutase (SOD), glutathione peroxidase (GPx), and catalase. In terms of anti-inflammation, melatonin inhibits the activation of NF-κB and activator protein-1 (AP-1), reducing pro-inflammatory cytokine production while promoting the production of anti-inflammatory cytokines like interleukin-10 (IL-10) (32). Moreover, melatonin can regulate autophagy in cardiomyocytes, helping to remove damaged organelles and proteins and maintain cellular homeostasis (33). Animal studies have shown that melatonin administration can improve cardiac function in sepsis-induced models, but its clinical application in SCM requires more extensive human trials.

#### 3.2.3 Metabolic modulators

##### 3.2.3.1 Metformin

Metformin, a first-line drug for type 2 diabetes, has potential in the treatment of SCM through multiple mechanisms (34). It activates the adenosine monophosphate-activated protein kinase (AMPK) pathway, promoting glucose uptake and utilization in cardiomyocytes, improving energy metabolism efficiency, and reducing lactate production (35). Additionally, metformin has anti-inflammatory and anti-oxidative effects, inhibiting NF-κB and MAPK signaling pathways and decreasing pro-inflammatory cytokine production. Metformin also increases the expression of antioxidant enzymes, scavenging ROS and reducing oxidative stress in cardiomyocytes (36). Moreover, metformin can improve endothelial function by promoting NO production through eNOS activation (37). Although pre-clinical studies have shown promising results, clinical evidence for metformin's use in SCM is limited, and more research is needed to determine optimal dosage and treatment duration (see Figure 1B).

#### 3.2.4 Other repurposed drugs

Other old drugs are also being explored for SCM treatment. Angiotensin-converting enzyme inhibitors (ACEIs) and angiotensin II receptor blockers (ARBs), commonly used in hypertension and heart failure treatment, may benefit SCM by modulating the renin-angiotensin-aldosterone system (RAAS) (38–40). They can reduce afterload, improve cardiac remodeling, and exert anti-inflammatory and antioxidant effects. Cyclosporine A, an immunosuppressant, has been studied for its potential to regulate the immune response in sepsis and reduce myocardial injury (68), though concerns about nephrotoxicity and other side effects need to be addressed.

### 3.3 Traditional Chinese medicine for the treatment of SCM

Traditional Chinese medicine offers a range of natural extracts and formulas that can effectively treat SCM by targeting inflammation, oxidative stress, apoptosis, and calcium handling. While most evidence remains pre-clinical, these TCM components align with the therapeutic targets identified in our mechanistic model. We present key examples, focusing on their proposed mechanisms of action.

#### 3.3.1 Emodin

Emodin primarily targets inflammation. It inhibits the nuclear factor-κB (NF-κB) signaling pathway, which reduces the production of pro-inflammatory cytokines such as TNF-α, IL-1β, and IL-6 (41). Emodin also has antioxidant effects, as it scavenges free radicals and up-regulates antioxidant enzymes, protecting cardiomyocytes from oxidative damage (42, 43). Moreover, it regulates the apoptotic process by preventing the release of cytochrome c from mitochondria and modulating the expression of anti-apoptotic and pro-apoptotic proteins (43), and protects mitochondrial function as a potential Sirt3 modulator (44) (see Figure 1C).

#### 3.3.2 Artemisinin

demonstrates significant anti-inflammatory and immunomodulatory effects in SCM. It modulates immune cell function, reducing the overactivation of macrophages and neutrophils. Artemisinin inhibits the production of pro-inflammatory cytokines and promotes the production of anti-inflammatory cytokines like IL-10 (45). Additionally, it scavenges ROS and protects mitochondrial function, ensuring sufficient energy production for cardiomyocytes (46). By targeting both inflammation and mitochondrial function, artemisinin addresses two key nodes in the SCM pathway (see Figure 1C).

#### 3.3.3 Shenfu injection (SFI)

SFI, is a classic TCM formulation that enhances microcirculation and reduces apoptosis via the AKT/GSK-3β pathway activation (47). Furthermore, SFI protects against sepsis-induced myocardial injury in mice through the suppression of myocardial apoptosis. It upregulates the protein expression of Bcl-2 and downregulates the protein expression of Bid, t-Bid and caspase-9, and alleviates sepsis-induced mitochondrial damage (48). SFI's effects are thus closely linked to the mitochondrial and cell death pathways (see Figure 1C).

#### 3.3.4 Caraway

Caraway (*Carum carvi*), a traditional medicinal and culinary herb, contains bioactive compounds that are beneficial in treating SCM. Its essential oil components, such as carvone and limonene, can inhibit the production of pro-inflammatory cytokines by suppressing the activation of NF-κB and MAPK signaling pathways in immune cells and cardiomyocytes. In addition, caraway extracts have strong antioxidant capabilities. They can scavenge free radicals, increase the activity of antioxidant enzymes, and reduce oxidative stress - induced damage to cardiomyocytes, protecting the heart from the harmful effects of sepsis-associated inflammation and oxidation.

#### 3.3.5 Curcumin

Curcumin mitigates inflammation and apoptosis through TLR1/NF-κB inhibition and improves mitochondrial dynamics in septic models. *Curcuma longa*, a leafy plant of the ginger family, is a common and safe compound with multiple pharmacological actions, including scavenging of oxygen free radicals, attenuation of inflammatory response, and antifibrotic effects. Great progress has been made in the study of sepsis-associated rodent models and in vitro cellular models. However, the evidence of curcumin in the clinical management practice of sepsis is still insufficient; hence, it is very important to systematically summarize the study of curcumin and sepsis pathogenesis.

### 3.4 New approaches in the treatment of SCM

#### 3.4.1 Anti-inflammatory therapy

The inflammatory response plays a crucial role in the pathogenesis of SCM. Corticosteroids, such as low-dose hydrocortisone, have been studied for their broad anti-inflammatory effects (49). They can reduce the production of pro-inflammatory cytokines and improve the body's stress response. However, their use in SCM is controversial due to the risk of secondary infections and uncertain clinical benefits, as reflected in clinical guidelines. Monoclonal antibodies against specific pro-inflammatory cytokines, such as anti-TNF-α, have also been investigated (50). Unfortunately, current clinical trial results have been unsatisfactory, highlighting the complexity and redundancy of the inflammatory network, which may not respond to single-cytokine blockade (see Figure 1D).

#### 3.4.2 Mitochondrial-targeted therapy

Mitochondrial dysfunction is a central feature of SCM. Mitochondrial-targeted antioxidants, like MitoQ, can accumulate in mitochondria, scavenge ROS, and protect mitochondrial membranes and functions (51). Pre-clinical studies have demonstrated that the administration of MitoQ improves myocardial function in animal models of SCM. Strategies to enhance mitochondrial biogenesis, such as using peroxisome proliferator-activated receptor-γ co-activator 1α (PGC-1α) agonists, are also being explored. PGC-1α promotes mitochondrial biogenesis and function, which may be beneficial in treating SCM (69). Rosmarinic acid and melatonin have been shown to activate Sirt1/PGC-1α and stabilize mitochondrial dynamics, respectively (70, 71) (see Figure 1D).

#### 3.4.3 Stem cell therapy

Stem cell therapy, particularly using mesenchymal stem cells (MSCs), has shown promise in treating SCM (52). MSCs have potent immunomodulatory and paracrine properties, secreting cytokines and growth factors like VEGF and IGF-1. These factors promote angiogenesis, inhibit apoptosis, and improve myocardial function. Animal studies have reported improved cardiac function and reduced myocardial fibrosis after MSC transplantation (53). However, several challenges remain, including the optimal source of stem cells, the best transplantation route, and long-term safety and efficacy (see Figure 1D).

#### 3.4.4 Other targeted interventions

##### 3.4.4.1 Pyroptosis inhibition

Excessive pyroptosis, a pro-inflammatory form of cell death, worsens myocardial injury. Agents like irisin and carvacrol attenuate SCM by suppressing NLRP3/GSDMD-mediated pyroptosis (72, 73) (see Figure 1D).

##### 3.4.4.2 Ferroptosis modulation

Iron-dependent cell death (ferroptosis) contributes to mitochondrial dysfunction in SCM. Resveratrol and dapagliflozin mitigate ferroptosis through the Sirt1/Nrf2 and GPX4 pathways, respectively (54, 74) (see Figure 1D).

## 4 Active small molecules for the treatment of SCM

As mentioned above, the treatment of SCM requires a multifaceted approach that targets these various aspects. Active small molecules, due to their specific biological activities, have emerged as promising therapeutic agents for the management of SCM. Several classes of active small molecules have been reported to have potential therapeutic value in the treatment of SCM.

**Figure 2:**
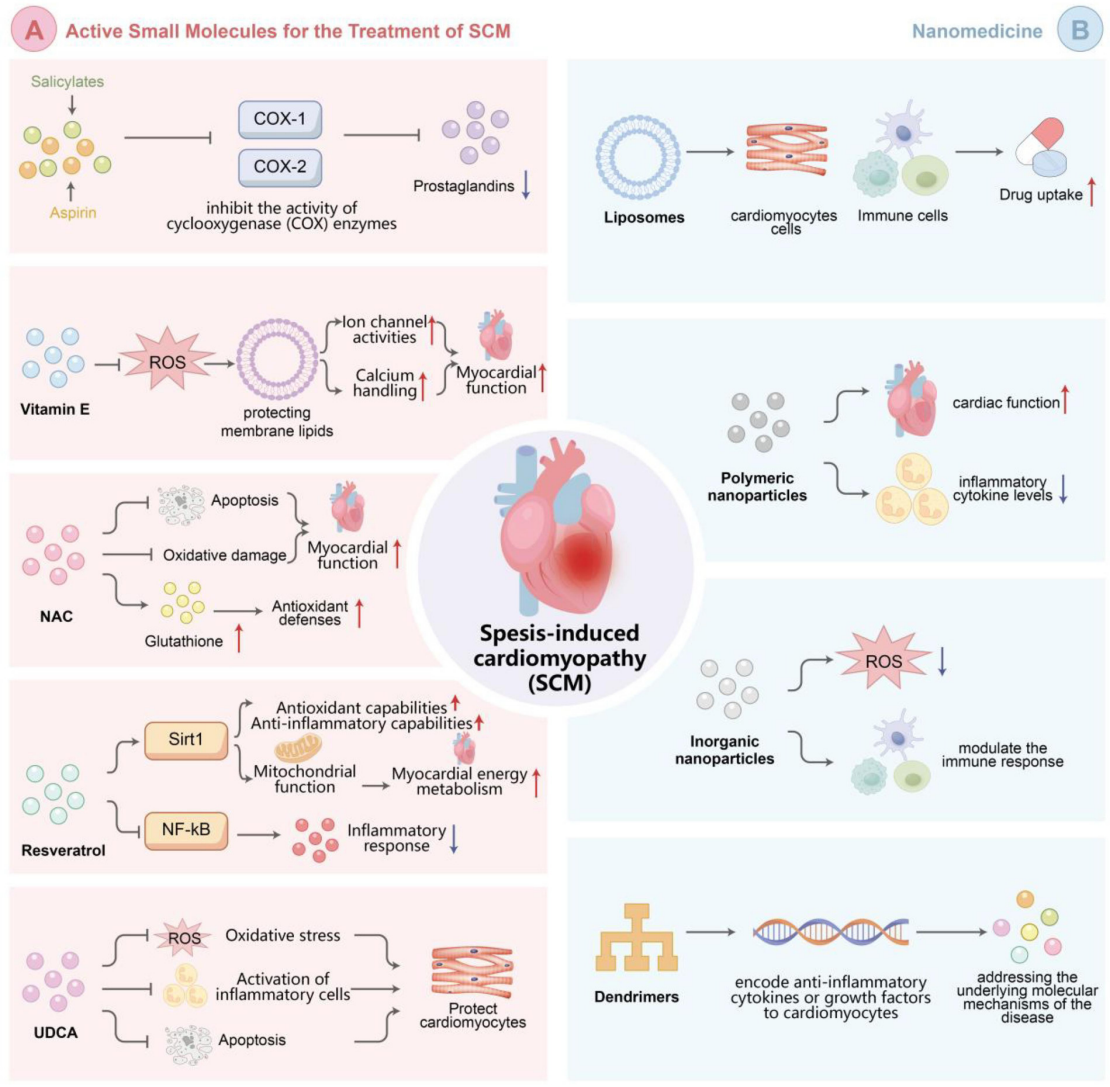
Active Small Molecules and nanomedicine for SCM treatment. (**A**) Active small molecules, such as salicylates,vitamin E, N-acetylcysteine (NAC),resveratrol, and ursodeoxycholic bile acids (UDCA), exert cardioprotective effects through antioxidant, anti-inflammatory, and anti-ferroptosis mechanisms. (**B**) Nanomedicine platforms, including liposomes, polymeric nanoparticles, inorganic nanoparticles (e.g., nanoceria) and dendrimers,enable targeted drug delivery and multifunctional therapeutic effects in septic cardiomyopathy.

Salicylates, exemplified by aspirin, possess well-established anti-inflammatory properties. They inhibit the activity of cyclooxygenase (COX) enzymes, particularly COX-1 and COX-2. By impeding the synthesis of prostaglandins, key inflammatory mediators, salicylates can mitigate the inflammatory response in sepsis. In the context of SCM, this anti-inflammatory action helps safeguard cardiomyocytes against inflammatory damage and may favorably impact myocardial contractility and cardiac function recovery (75) (see Figure 2A).

Vitamin E is a lipid-soluble antioxidant that inserts into cell membranes, protecting membrane lipids from peroxidation by eliminating lipid-peroxyl radicals. In SCM, oxidative stress-induced lipid peroxidation of cardiomyocyte membranes can disrupt membrane fluidity and function, influencing ion channel activities and calcium handling (76). Vitamin E prevents this peroxidation process, upholds the normal structure and function of cardiomyocyte membranes, and consequently contributes to the preservation of myocardial contractility and relaxation function (77) (see Figure 2A).

N-acetylcysteine (NAC) is a precursor of glutathione, a pivotal endogenous antioxidant. It elevates the intracellular level of glutathione, fortifying the body's antioxidant defenses. Glutathione reacts with ROS, transforming them into harmless substances and maintaining cellular redox balance. In SCM, NAC shields cardiomyocytes from oxidative damage, curtails apoptosis, and boosts cardiac function by augmenting the antioxidant defense system (57) (see Figure 2A).

Resveratrol, a natural polyphenol present in grapes and other plants, demonstrates diverse biological activities. It can activate the Sirt1 pathway, which is implicated in cell metabolism, stress resistance, and longevity (55). In SCM, resveratrol-induced Sirt1 activation can strengthen the antioxidant and anti-inflammatory capabilities of cardiomyocytes, improve mitochondrial function, and regulate myocardial energy metabolism (56). Moreover, resveratrol can inhibit the activation of NF-κB and other inflammatory signaling pathways, attenuating the inflammatory response in the myocardium (see Figure 2A).

Ursodeoxycholic bile acids (UDCA) is a potential cardioprotective small molecule. It can adjust the function of cell membranes, enhance mitochondrial function, and exhibit anti-inflammatory and anti-apoptotic effects (78). In SCM, UDCA may protect cardiomyocytes by reducing oxidative stress, inhibiting the activation of inflammatory cells, and preventing apoptosis, thereby contributing to the maintenance of cardiac function (see Figure 2A).

## 5 Nanomedicine: a promising avenue for SCM treatment

Nanomedicine offers promising prospects for the treatment of SCM due to its unique characteristics, such as high surface-to-volume ratio, customizable physicochemical properties, and the ability to encapsulate diverse therapeutic agents (58). This allows for enhanced drug bioavailability and targeted delivery to the diseased myocardium, overcoming the limitations of conventional drugs.

Liposomes, made of phospholipid bilayers, can encapsulate both hydrophilic and hydrophobic drugs, protecting them from premature degradation and enhancing drug uptake by cardiomyocytes and immune cells (59). Targeted liposomes, with attached ligands like antibodies or peptides, can specifically bind to receptors overexpressed in the septic myocardium, increasing local drug concentration and reducing side effects (60, 61) (see Figure 2B).

Polymeric nanoparticles, synthesized from natural or synthetic polymers, offer controlled drug release and can be engineered for multifunctionality, loading multiple drugs and responding to specific stimuli in the septic microenvironment (62). Pre-clinical studies have validated their potential in improving cardiac function, reducing myocardial fibrosis, and decreasing inflammatory cytokine levels in animal models of SCM (63) (see Figure 2B).

Inorganic nanoparticles, such as cerium oxide nanoparticles (nanoceria), have inherent antioxidant and anti-inflammatory properties, and can also facilitate imaging-guided therapy by acting as MRI contrast agents (64–66). They can effectively scavenge ROS in the myocardium and modulate the immune response (67) (see Figure 2B).

Dendrimers, highly branched macromolecules, can precisely load drugs within their cavities or on their surface, and can be functionalized with targeting ligands for specific delivery (79). They also show potential in gene therapy for SCM, delivering genes that encode anti-inflammatory cytokines or growth factors to cardiomyocytes, addressing the underlying molecular mechanisms of the disease (80) (see Figure 2B).

## 6 Towards phenotype-guided therapy in SCM

A major challenge in managing SCM is the heterogeneity of its clinical presentation. Patients can present with different hemodynamic profiles, which may require distinct therapeutic approaches.

### 6.1 High-output state (vasodilatory shock with preserved or hyperdynamic cardiac function)

In these patients, the primary problem is profound vasodilation. Treatment focuses on vasopressors (e.g., norepinephrine) to restore peripheral vascular tone. Therapies that increase cardiac contractility (e.g., dobutamine) might be unnecessary or even harmful by increasing myocardial oxygen demand.

### 6.2 Low-output state (overt septic cardiomyopathy)

This phenotype is characterized by a significant drop in cardiac output and ejection fraction. Here, the focus shifts to improving cardiac contractility. In addition to standard inotropes, therapies targeting mitochondrial function (e.g., levosimendan) or modulating inflammation (e.g., β-blockers to reduce tachycardia and improve filling) may be particularly beneficial.

Right ventricular failure: Sepsis can also lead to acute right ventricular dysfunction, often exacerbated by increased pulmonary vascular resistance from acute respiratory distress syndrome (ARDS). Management in these cases may require specific strategies to reduce right ventricular afterload and support its function.

### 6.3 Future biomarker-guided therapy

Emerging biomarkers, such as specific circulating miRNAs or exosomal content, may 1 day help identify the dominant pathological pathway in an individual patient (e.g., “inflammatory phenotype” vs. “mitochondrial phenotype”), allowing for the selection of the most appropriate targeted therapy (e.g., an anti-inflammatory drug vs. a mitochondrial protectant).

## 7 Conclusion and prospectives

In conclusion, the current clinical management of SCM, as reflected in guidelines such as the Surviving Sepsis Campaign, relies on a multifaceted supportive approach. This includes prompt infection control with antibiotics and source removal, judicious fluid resuscitation, and the use of vasopressors (e.g., norepinephrine) and inotropic agents (e.g., dobutamine, levosimendan) to correct hemodynamic instability. While these interventions are essential for patient survival, they primarily address the systemic consequences of sepsis rather than the specific molecular drivers of myocardial depression, leaving a significant gap in promoting true cardiac recovery.

The multifactorial pathogenesis of SCM underscores the need for targeted therapeutic approaches beyond this conventional supportive care. The research strategies discussed in this review offer a spectrum of potential options, each with distinct advantages and disadvantages regarding their translation to clinical practice.

Repurposing established drugs like statins, β-blockers, and metformin presents a cost-effective and readily translatable strategy due to their well-characterized safety profiles and known pharmacokinetics. Their primary advantage lies in their pleiotropic effects, modulating inflammation, oxidative stress, and energy metabolism. However, their main disadvantage is that these effects are often indirect and not specifically designed for the septic heart, potentially leading to limited efficacy or off-target effects. The heterogeneity of clinical results to date suggests that identifying the right patient subgroup and optimal timing for these drugs is critical. Similarly, components of Traditional Chinese Medicine (TCM), such as emodin and artemisinin, offer unique multi-target advantages by regulating pathways like NF-κB and mitochondrial function. Their major hurdle for widespread clinical adoption, particularly in Western medicine, remains the lack of rigorous, large-scale, randomized controlled trials to standardize formulations and validate efficacy and safety.

Newer, more targeted approaches are also on the horizon. Mitochondrial-targeted therapies (e.g., MitoQ) and specific cell-death inhibitors (targeting pyroptosis or ferroptosis) offer the advantage of high mechanistic specificity, which could translate into potent effects with fewer systemic side effects. However, they are still largely in the pre-clinical phase, and their clinical development is costly and complex. Nanomedicine-based drug delivery systems could revolutionize SCM treatment by enhancing drug bioavailability and enabling targeted delivery to the myocardium, thereby improving efficacy and reducing toxicity. Despite their immense promise, the complexity of their manufacturing, potential for unforeseen toxicity, and high regulatory barriers mean that their clinical application is likely further in the future.

Considering the balance of evidence and the practicality of implementation, repurposed drugs and certain well-studied TCM formulations (like Shenfu Injection) are arguably the closest to being integrated into clinical practice. They can leverage existing safety data and may be tested in pragmatic clinical trials in the near term. In contrast, while highly promising, strategies like stem cell therapy and advanced nanomedicines will require significantly more pre-clinical optimization and rigorous safety evaluations before they can be considered for human trials.

Future research should therefore prioritize large-scale, multicenter clinical trials to validate the efficacy and safety of the most promising repurposed drugs and TCM formulations. These trials should integrate precision medicine strategies, using patient phenotyping and biomarker-guided treatment to identify those most likely to benefit. Ultimately, a multimodal approach that combines conventional supportive care with targeted therapies—carefully selected based on a patient's individual pathophysiological profile—holds the potential to revolutionize SCM treatment, improving not just survival, but also meaningful cardiac function recovery.
